# Multiple FadD Acyl-CoA Synthetases Contribute to Differential Fatty Acid Degradation and Virulence in *Pseudomonas aeruginosa*

**DOI:** 10.1371/journal.pone.0013557

**Published:** 2010-10-21

**Authors:** Yun Kang, Jan Zarzycki-Siek, Chad B. Walton, Michael H. Norris, Tung T. Hoang

**Affiliations:** 1 Department of Microbiology, University of Hawaii at Manoa, Honolulu, Hawaii, United States of America; 2 Department of Molecular Biosciences and Bioengineering, University of Hawaii at Manoa, Honolulu, Hawaii, United States of America; 3 Department of Medicine, University of Hawaii at Manoa, Honolulu, Hawaii, United States of America; National Institutes of Health, United States of America

## Abstract

A close interconnection between nutrient metabolism and virulence factor expression contributes to the pathophysiology of *Pseudomonas aeruginosa* as a successful pathogen. *P. aeruginosa* fatty acid (FA) degradation is complicated with multiple acyl-CoA synthetase homologs (FadDs) expressed *in vivo* in lung tissue during cystic fibrosis infections. The promoters of two genetically linked *P. aeruginosa fadD* genes (*fadD1* and *fadD2*) were mapped and northern blot analysis indicated they could exist on two different transcripts. These FadDs contain ATP/AMP signature and FA-binding motifs highly homologous to those of the *Escherichia coli* FadD. Upon introduction into an *E. coli fadD* ^-^/*fadR* ^-^ double mutant, both *P. aeruginosa fadD*s functionally complemented the *E. coli fadD* ^-^/*fadR* ^-^ mutant, allowing degradation of different chain-length FAs. Chromosomal mutagenesis, growth analysis, induction studies, and determination of kinetic parameters suggested that FadD1 has a substrate preference for long-chain FAs while FadD2 prefers shorter-chain FAs. When compared to the wild type strain, the *fadD2* mutant exhibited decreased production of lipase, protease, rhamnolipid and phospholipase, and retardation of both swimming and swarming motilities. Interestingly, *fadD1* mutant showed only increased swarming motility. Growth analysis of the *fadD* mutants showed noticeable deficiencies in utilizing FAs and phosphatidylcholine (major components of lung surfactant) as the sole carbon source. This defect translated into decreased *in vivo* fitness of *P. aeruginosa* in a BALB/c mouse lung infection model, supporting the role of lipids as a significant nutrient source for this bacterium *in vivo*.

## Introduction

**Figure 1:**
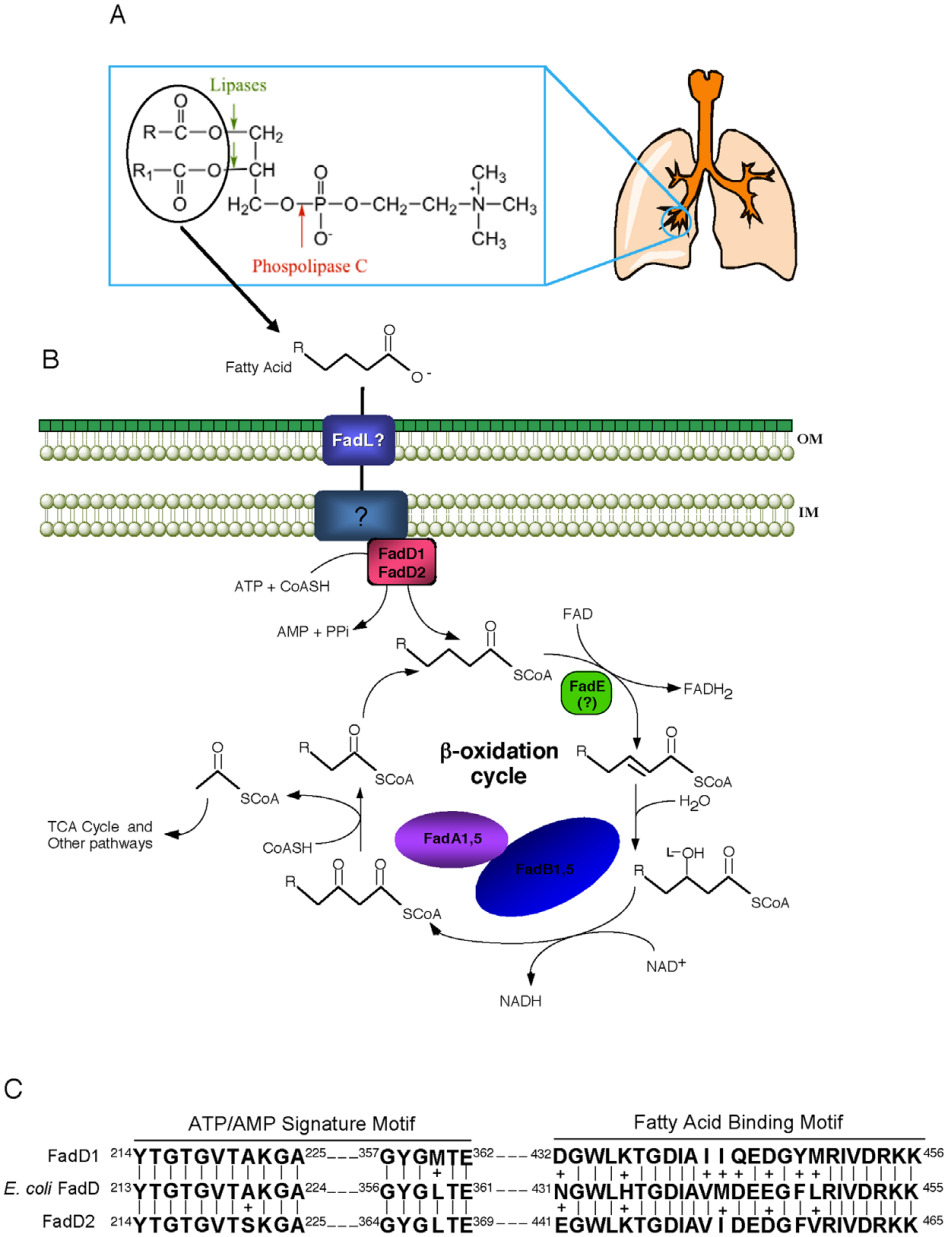
The proposed FA degradation pathway in *P. aeruginosa* based on *E. coli* β-oxidation. (A) Phosphatidylcholine (PC) is the major component of lung surfactant. PC can be cleaved by *P. aeruginosa* phospholipase C and lipases producing free fatty acids that are degraded via the β-oxidation pathway by this bacterium. (B) FAs are transported through the outer membrane aided by an unidentified *P. aeruginosa* FadL [13], [67]. In *E. coli*, FA may be transported through the inner membrane via an unknown mechanism coupled to a single peripheral membrane FadD protein [18]. However, *P. aeruginosa* contains at least two FadDs (FadD1 and FadD2). Although there are over a dozen potential FadE homologues in the *P. aeruginosa* genome, the specific enzyme(s) that catalyzes this reaction has not been identified. FadB catalyzes the next two steps followed by cleavage of the 3-keto-acyl-CoA by FadA. Two *fadBA* operons (*fadBA1* and *fadBA5*) have been identified in *P. aeruginosa* [29], [30]. (C) Alignment of the *P. aeruginosa* FadD1 and FadD2 ATP/AMP-signature and FA-binding motifs with the FadD motifs of *E. coli* [18], [19]. Abbreviation for Fad-proteins: FadA, 3-ketoacyl-CoA thiolase; FadB, enoyl-CoA hydratase and 3-hydroxyacyl-CoA dehydrogenase; FadD, acyl-CoA synthetase; FadE, acyl-CoA dehydrogenase; FadL, outer membrane FA translocase.

To occupy a diverse range of ecological niches, *Pseudomonas aeruginosa* must evolve and maintain a wide array of metabolic pathways for nutrient uptake and utilization. This adaptive flexibility allows *P. aeruginosa*, a ubiquitous Gram-negative saprophyte, to occupy environmental niches in both soil and water and to transition into a potentially pathogenic lifestyle with humans, plants, animals, and other microbes [1]–[4]. This bacterium has been responsible for a myriad of infections including serious bacteremia and nosocomial pneumonia [5]–[8], and it has been shown to be the major cause of morbidity and mortality among cystic fibrosis (CF) patients aged 18–24 years [9], [10]. *P. aeruginosa* thrives both environmentally and within a human host because of its extensive repertoire of virulence factors (e.g., LasA/LasB and alkaline proteases, phospholipases, lipases, exotoxin A, type III secretion exoenzymes S/T/U/Y, rhamnolipid, alginate biofilm, hydrogen cyanide synthesis, and others) and its capacity to metabolize 70–80 different organic substrates as sole carbon sources, notably different chain-length fatty acids (FA, C_4_–C_18_) [11]. Our previous work suggested that *P. aeruginosa* expresses phospholipases and lipases *in vivo* that degrades phosphatidylcholine (PC; Fig. 1A) as a nutrient source for bacterial replication in the lungs of CF patients [12]. In support of these results, Miller et al. [13] have shown that *P. aeruginosa* utilizes type IV pili to twitch towards phospholipids (i.e. phosphatidylethanolamine and PC) and long-chain FA (LCFAs).

Fatty acid degradation (Fad) in the model microbe, *Escherichia coli*, employs enzymes of the Fad pathway encoded by the *fad*-regulon [14], [15]. *E. coli* possesses a single FadD, a 62-kDa fatty acyl-CoA synthetase (FACS or AMP-forming fatty acid:CoA ligase), encoded by the *fadD* gene [16], [17]. The FadD protein possesses two highly conserved sequence elements corresponding to a proposed ATP/AMP signature motif [17], [18], as well as a signature motif involved in FA substrate binding and specificity [19] (Fig. 1C). Following FadL-mediated importation of exogenous FAs through the outer membrane [20]–[22] and an unknown transportation process through the inner membrane, FadD appears to employ these two motifs to activate FAs in a two-step process [18], [19]. In the first step of activation, an acyl bond between the α-phosphoryl group of an ATP and the carboxyl group of a FA is formed creating a fatty acyl-adenylate intermediate and releasing pyrophosphate. In the second step, the release of AMP occurs concomitantly during thioester bond formation between the fatty acyl group and the sulfhydryl group of coenzyme A in the second step [23]. This FadD-catalyzed reaction produces fatty acyl-CoA, a molecule capable of degradation by the β-oxidation cycle or exerting transcriptional control on the *E. coli fad*-regulon by interacting with the FadR regulator to derepress *fad*-genes [24]–[27]. However, it seems currently that some *fad*-genes in *P. aeruginosa* are induced, not by fatty acyl-CoA, but by LCFAs [24]–[28]. While the biochemistry and physiology of FadD have been well characterized in *E. coli*, relatively little is known about FadD(s) in *P. aeruginosa*. The *P. aeruginosa* β-oxidation cycle in Fad has only been partially characterized with respect to FadBAs (Fig. 1B) [29], [30]. Fad enzymes, including the broad substrate specificity of the FACS, have also been characterized in *Pseudomonas fragi* [31]–[33]. A study on *Pseudomonas putida* originally isolated and characterized one FACS with a broad substrate range [34]. Additional work further characterized the role of this *P. putida* enzyme and identified a second FACS, naming them FadD1 and FadD2, respectively [35], [36]. In this dual FadD system, it was shown that FadD1 played a dominant role in FA metabolism while FadD2 was activated only when FadD1 was inactivated [36]. Comparison of the significantly larger size of the *Pseudomonas* genome relative to that of *E. coli*, such genetic redundancies are not unexpected. However, the importance of the redundancy and functions of these enzymes in Fad are uncertain.

Studies on other species have indicated a link between FACS, nutrient metabolism, and the expression of virulence factors [37]–[43]. In *Mycobacterium tuberculosis*, 36 *fadD* homologues were identified [39]. A null mutation in the *M. tuberculosis fadD28* gene showed significant replication restriction in mouse lungs, as a result of defects in cell-wall biosynthesis and the production of complex lipids [40]. In addition, *fadD33* in the *M. tuberculosis* H37Ra strain was shown to play a role in supporting growth in mouse livers [42]. Similar to these *Mycobacterium* studies, the use of random transposon mutagenesis has led to the isolation of a *fadD* mutant in *Salmonella enterica* serovar Typhimurium which was shown to reduce the expression of *hilA* (a proposed transcriptional activator of genes in the type III secretion system [38]) and invasion genes three- to five-fold [41]. A *Xanthomonas campestris fadD* homolog *rpfB* mutant has decreased production of protease, endoglucanase, and polygalacturonate lyase due to the inability to generate a diffusible extracellular factor containing a FA moiety [37]. A Tn5 insertion in the *fadD* gene of *Sinorhizobium meliloti* displayed an increased swarming phenotype compared to wildtype, resulting in an observed decrease in alfalfa root nodulation [43]. Many of these studies correlated *fadD* mutations with decreased virulence, but did not confirm or elucidate its enzymatic role in FA metabolism. We have previously shown that *P. aeruginosa* expresses *fadD1* and *fadD2* (PA3299 and PA3300) during lung infections in CF patients, suggesting the importance of Fad in lipid nutrient acquisition *in vivo* [12]. However, the role of *fadD* on virulence and growth of the bacteria *in vivo* has not been characterized.

Here, we characterized the FadD1 and FadD2 (PA3299 and PA3300) and the respective genes with relevance to their biochemistry and the effect on *P. aeruginosa* pathophysiology. The results of genetic analyses and biochemical characterization provided insight into reasons why redundancies in *fadD* are beneficial to this pathogen. Interestingly, *fadD* mutants displayed alterations in swimming and swarming motility and the production of lipases, phospholipases, rhamnolipids, and proteases. The *fadD* mutants with reduced ability to grow on phosphatidylcholine as a sole carbon source showed decreased fitness in a mouse lung infection model. These results provide the initial characterization of *P. aeruginosa fadD* genes and suggest a pathophysiological link between Fad and virulence.

## Results

### Comparison of two *fadD*s in *P. aeruginosa*

**Figure 2:**
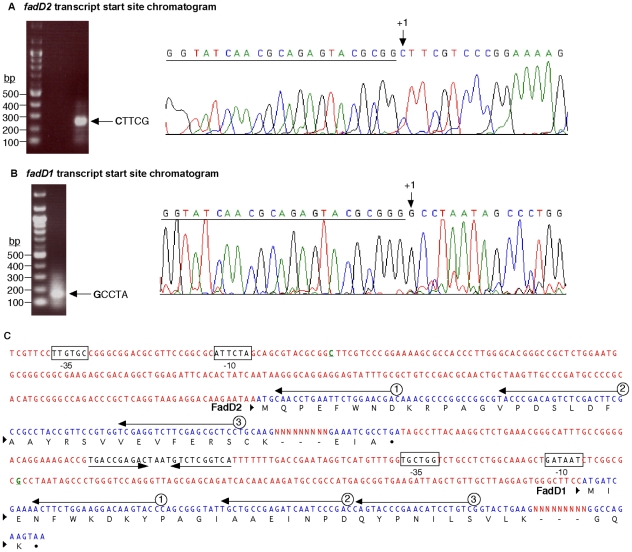
SMART mapping of the transcriptional start sites for *fadD2* and *fadD1*. (A) One SMART product was observed after PCR amplification of the cDNA with SMART and *fadD2* primers (oligonucleotides #798 and #373). Sequencing of the single band with a nested *fadD2* primer (oligonucleotide #374) displayed a reverse-complement sequence chromatogram, showing the *fadD2* transcriptional start site (indicated by +1 at the **C**TTCG sequence) and the underlined SMART primer sequence. (B) Likewise, the downstream *fadD1* transcriptional start site was mapped (at the **G** of the sequence **G**CCTA) by sequencing a single PCR product. (C) *fadD2* and *fadD1* coding sequences and the predicted −10 and −35 promoter regions are indicated (boxed). The intergenic region between *fadD2* and *fadD1* contains a potential transcriptional terminator or attenuator sequence (inverted arrows). For each gene, three black arrows indicate primers 1, 2 and 3 (#372/#375, #373/376, and #374/377) used for mapping *fadD2* and *fadD1*. Dashed lines indicate missing protein sequences and dots indicate stop codons.

Our previous work showed that two *P. aeruginosa fadD*s (*fadD1* and *fadD2*) were expressed *in vivo* during lung infection in CF patients [12]. FadD1 (PA3299) and FadD2 (PA3300) are 72% similar (54% identical) and 72% similar (53% identical) to the *E. coli* FadD, respectively, while *P. aeruginosa* FadD1 and FadD2 are 76% similar (60% identical) to each other. In addition, *fadD*1 and *fadD*2 are adjacent genes, separated by 234-bp and a possible Rho-independent transcriptional terminator (Fig. 2C). Convincingly, the ATP/AMP signature and FA-binding motifs described for the *E. coli* FadD are highly conserved in both *P. aeruginosa* FadD1 and FadD2 (Fig. 1C). This preliminary analysis suggests that *fadD1* and *fadD2* are both involved in Fad.

To confirm that *fadD1* and *fadD2* are important for Fad, we complemented the *E. coli fadD* ^-^/*fadR* ^-^ strain (E2011) with these *P*. *aeruginosa* genes. This *E. coli* strain contains a mutation in the *fadR* gene (*fadR* ^-^), the main repressor of the *fad*-regulon in *E. coli*, allowing the constitutive expression of other *fad*-genes of the aerobic Fad-pathway [24]–[27]. Both *fadD1* and *fadD2* of *P. aeruginosa* were able to functionally complement the *E. coli fadD^-^* mutant (Table 1). The *E. coli* K12 wildtype strain was able to metabolize C_12:0_ to C_18:1_^Δ9^ as expected, because long-chain acyl-CoA (≥C_12:0_) binds efficiently to FadR to induce the *fad*-regulon. Complementation of strain E2011 with the *E. coli fadD* gene (*fadD_EC_*) on plasmid pET15b resulted in growth similar to that of *E. coli* K12 on C_12:0_ to C_18:1_^Δ9^, as well as on C_10:0_ because of the deregulated *fad*-regulon as previously observed [44]. Both *P. aeruginosa fadD*s were able to complement the *E. coli fadD^-^*/*fadR^-^* strain to a similar level as the *fadD_EC_* complement (Table 1), suggesting that both FadD1 and FadD2 could activate LCFAs and medium-chain FAs (MCFAs). The various complements did not grow on short-chain FAs (SCFAs), suggesting that other *E. coli* Fad-enzymes may not support growth on SCFAs [45] and not necessarily that the *Pseudomonas* FadD proteins are incapable of producing short-chain fatty acyl-CoAs. The E2011 control strains, either with or without the pET15b empty vector, showed no growth on any FA (Table 1).

**Table 1:** Complementation of the *E. coli fadD* mutant with *P. aeruginosa fadD* homologues.

		Growth on different FAs and casamino acids^a^								
Strain	Plasmid	C_4:0_	C_6:0_	C_8:0_	C_10:0_	C_12:0_	C_14:0_	C_16:0_	C_18:1_^Δ9^	CAA
K12 (wildtype)	none	-	-	-	-	+4	+5	+4	+5	+6
E2011 (*fadD^-^ fadR* ^-^)	none	-	-	-	-	-	-	-	-	+6
E2011 (*fadD^-^ fadR* ^-^)	pET15b	-	-	-	-	-	-	-	-	+6
E2011 (*fadD^-^ fadR* ^-^)	pET15b-*fadD_Ec_*	-	-	-	+2	+3	+3	+4	+4	+6
E2011 (*fadD^-^ fadR* ^-^)	pET15b-*fadD1*	-	-	-	+2	+4	+4	+4	+4	+6
E2011 (*fadD^-^ fadR* ^-^)	pET15b-*fadD2*	-	-	-	+2	+4	+3	+4	+4	+6

a (-) denotes no growth on a patch; (+) denotes growth: (+1) is very little growth and (+6) is heavy growth after 3 days.

### *fadD2* and *fadD1* exist on two transcripts and are induced by FA of different lengths

**Figure 3:**
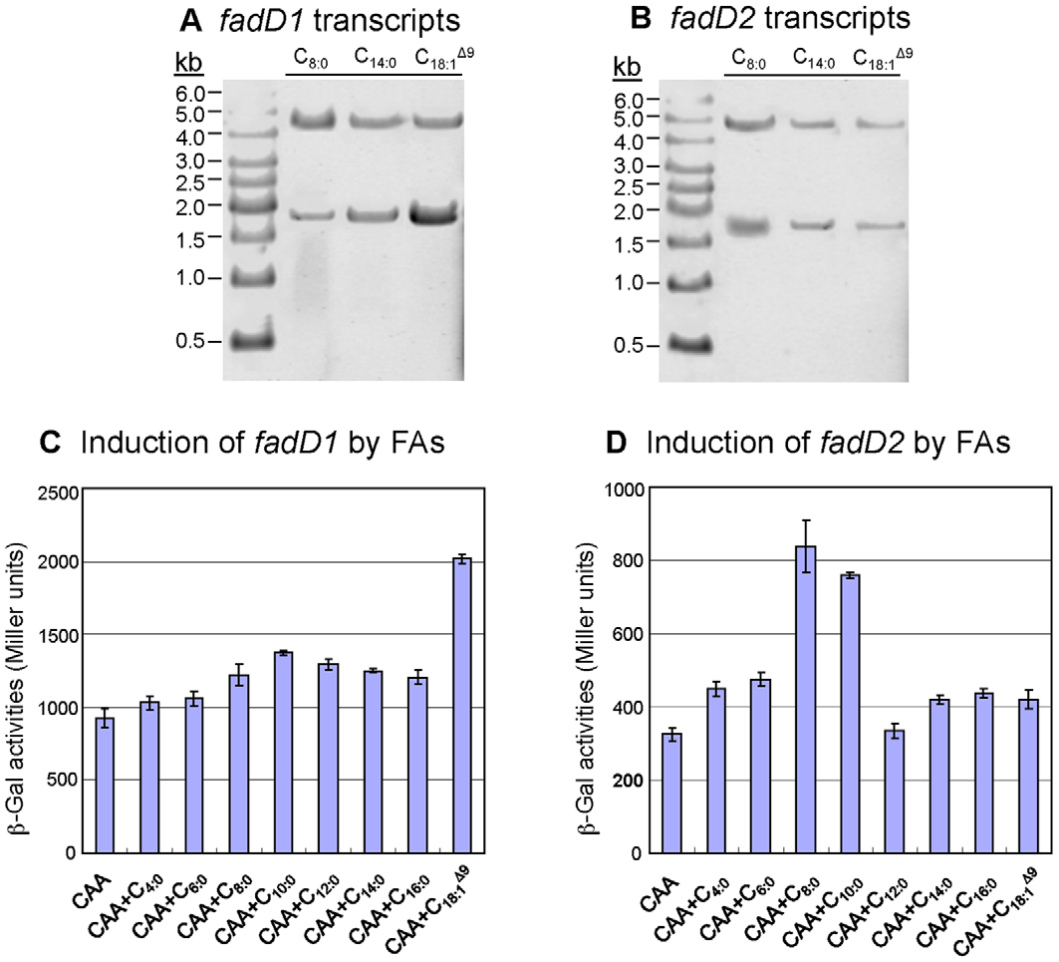
Transcriptional profile of *fadD1* and *fadD2* in various FAs. For a short- (C_8:0_), medium- (C_10:0_), and long-chain FA (C_18:1_^Δ9^), northern blot analysis indicated two possible transcripts for both *fadD* genes when probed with either *fadD1* (A) or *fadD2* (B). Gene-fusion studies of strains P518 (P*_fadD1_-lacZ*) and P520 (P*_fadD2_-lacZ*), grown to mid-log phase, showed differential expression of *fadD1* and *fadD2* in the presence of different FAs (C and D). (C) *fadD1* was up-regulated in the presence of the unsaturated LCFA (C_18:1_^Δ9^), while *fadD2* expression was significantly increased in the presence of shorter chain FAs (C_8:0_, C_10:0_) (D). For (C) and (D), all cultures had identical growth-rates and overall cell densities (data not shown).

To gain information on the regulatory regions of the *P. aeruginosa fadD*s, we mapped their transcriptional start sites to assign putative promoter sequences, and then determined transcription levels of each gene on various carbon sources (Figs. 2 and 3). Promoter mapping experiments indicated that each *fadD* had an independent transcriptional start site, suggesting that they were independently transcribed; however, northern blot analyses indicated that *fadD2* and *fadD1* can be co-transcribed on a single larger transcript or as smaller independent transcripts (Fig. 3A and 3B). Both *fadD2* and *fadD1* can exist as two different transcripts, suggesting some level of regulation by the predicted transcriptional terminator or attenuator sequence within the intergenic region (Fig. 2C). From Figure 3, we hypothesize that the promoter upstream of *fadD2* drives the expression of both genes, and the intergenic terminator attenuates the larger *fadD1* transcript. The *fadD1* promoter immediately downstream of this regulatory element was induced by LCFA (e.g. C_18:1_^Δ9^), and initiated the expression of the smaller *fadD1* transcript (Fig. 2C, 3A and 3B). Presumably, when there was no termination of transcription from the *fadD2* promoter, *fadD2* and *fadD1* were transcribed together on the larger transcript of the same size observed on both blots (Fig. 3A and 3B). Based on the determination that these *fadD* genes could be independently transcribed or co-transcribed, it was necessary to determine which chain-length FA induced *fadD1* and *fadD2*. The observed levels of induction showed that *fadD1* was mainly induced by LCFA, particularly C_18:1_^Δ9^, while *fadD2* was specifically induced by short- to medium-chain FA (Fig. 3C and 3D). Both *fadD* genes showed some level of expression under all conditions tested, indicating low levels of constitutive expression.

### *fadD* mutants showed reduced ability to grow on various FAs

**Figure 4:**
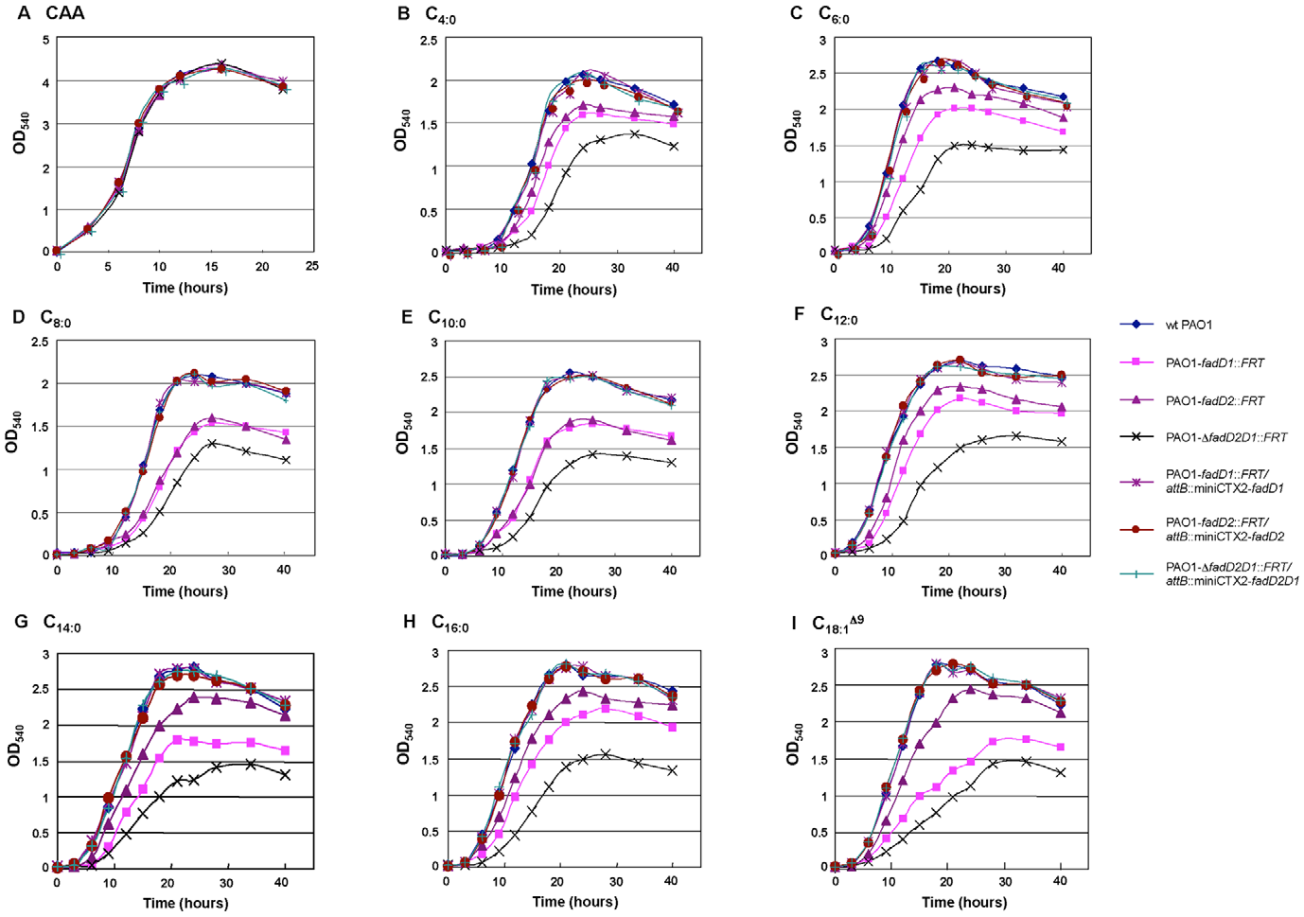
Growth analysis of *fadD* mutants using various FAs as sole carbon sources. Although *fadD* mutants showed various defects when grown with FAs of different chain-lengths (see top of graphs in B-I), no growth defects were observed for any of the mutants when grown with casamino acids (CAA) as a control (A). All three *P. aeruginosa* mutants were fully complemented by the respective missing gene(s) and grew as well as the wildtype PAO1 on all carbon sources. Not shown are the three control mutant strains (PAO1-*fadD1*::*FRT*/*attB*::miniCTX2, PAO1-*fadD2*::*FRT*/*attB*::miniCTX2, and PAO1-Δ*fadD2D1*::*FRT*/*attB*::miniCTX2) having the empty miniCTX2 integrated into their chromosomes, where all had similar growth characteristics to the non-complemented mutants.

To further confirm the involvement of each *fadD* in Fad, we generated single and double mutants for growth analysis on various FAs as sole carbon sources (Fig. 4). As previously observed for *fadBA* mutants [30], growth defects were exemplified by slower growth and lower overall final cell densities in various FA media, presumably, due to reduced rates of Fad and growth inhibiting intermediates. Both *fadD1* and *fadD2* single mutants had various levels of defects when grown on all FAs (Fig. 4). However, the *fadD1* mutant displayed a greater growth defect on all FAs than the single *fadD2* mutant, with the exception of C_8:0_ and C_10:0_ where FadD2 seems to be equally as important as FadD1 (Fig. 4D and 4E). The Δ*fadD2D1* mutant showed more dramatic growth defects on all FAs than the individual single mutants, indicating that both proteins were involved in the metabolism of all chain-length FAs tested. The lack of a complete defect in Fad of this double mutant suggests that other *fadDs* exist in *P. aeruginosa*. The complemented single and double mutants fully restored growth on all FAs (Fig. 4), while empty vector miniCTX2 controls did not complement growth on the FAs (data not shown). No apparent defects were observed for any mutant grown with casamino acids (CAA) as a sole carbon source (Fig. 4A). Based on the physiological data (Fig. 3 and 4), *fadD1* was found to be important for the metabolism of all FAs, particularly the unsaturated LCFA oleate, while *fadD2* was also important in Fad but more so for MCFA (C_8:0_ and C_10:0_) degradation.

### Kinetic properties of purified FadDs support differential FA chain-length preferences

**Figure 5:**
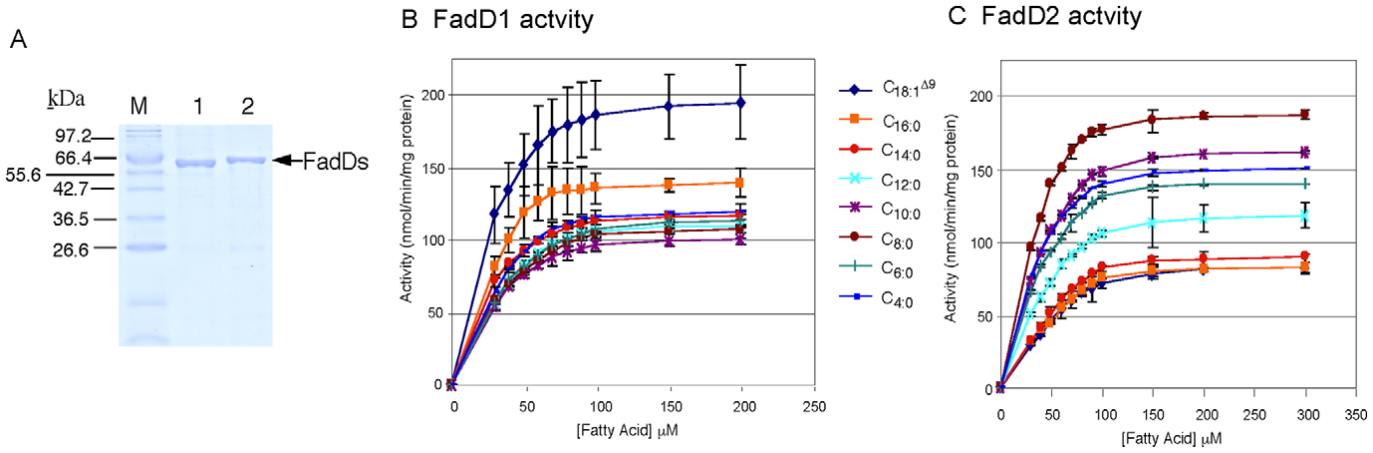
Purification and biochemical characterization of the two *P. aeruginosa* FadDs. (A) FadD1 (lane 1; MW = 61,655) and FadD2 (lane 2; MW = 61,737) were purified to near homogeneity from an *E. coli fadD* ^-^ strain to prevent potential contamination of *E. coli* FadD. FadD1 activities (B) were higher for LCFAs (C_18:1_^Δ9^>C_16:0_), while FadD2 (C) had higher activities for shorter chain FAs (C_8:0_>C_10:0_>C_4:0_>C_6:0_).

To determine the substrate specificities of each FadD and further clarify the reason why *P. aeruginosa* possesses multiple *fadD* homologues, both FadD proteins were purified to near homogeneity from an *E. coli fadD* ^-^ strain to ensure that all acyl-CoA synthetase activities were derived only from purified recombinant FadD1 or FadD2 (Fig. 5A). FadD1 of *P. aeruginosa* coupled CoASH to LCFA better than to SCFA or MCFA, as exemplified by larger *V*_max_ and lower *K_m_* values for C_18:1_^Δ9^ and C_16:0_ than FAs of other chain-lengths (Fig. 5B and Table 2). The reverse was true for FadD2, where this enzyme had higher *V*_max_ and lower *K_m_* for SCFA and MCFA than LCFA (Fig. 5C and Table 2). The catalytic efficiency (*k*_cat_/*K_m_*) of FadD1 was significantly higher for LCFA (C_18:1_^Δ9^, C_16:0_, and C_14:0_) than MCFA (C_12:0_ to C_8:0_) or SCFA (C_6:0_ and C_4:0_), while the catalytic efficiency of FadD2 was higher for MCFA and SCFA than LCFA (Table 2). The kinetic parameters for ATP and catalytic efficiency of both enzymes were comparable when ATP was limited in the reaction, with FadD1 being a slightly better catalytic enzyme for ATP than FadD2 (Table 2). Clearly, multiple FadDs in *P. aeruginosa*, with broad substrate conversion capabilities and overlapping chain-length preferences, afford this bacterium the ability to optimally metabolize FAs of various chain-lengths.

**Table 2:** Kinetic properties of FadD1 and FadD2 with various substrates.

	FadD1 Kinetic Parameter^a^				FadD2 Kinetic Parameter^a^			
Substrate varied	*V*_max_ b	*k*_cat_ c	*K_m_* d	*k*_cat_ */K_m_* e	*V*_max_ b	*k*_cat_ c	*K_m_* d	*k*_cat_ */K_m_* e
ATP	213	0.219	10.6	20.7	182	0.187	10.9	17.2
C_4:0_	137	0.141	27.4	5.1	167	0.172	33.3	5.2
C_6:0_	133	0.137	26.7	5.1	159	0.164	31.8	5.2
C_8:0_	125	0.128	25.0	5.1	204	0.210	20.4	10.3
C_10:0_	116	0.119	23.3	5.1	182	0.187	36.4	5.1
C_12:0_	130	0.134	26.0	5.1	137	0.141	41.1	3.4
C_14:0_	130	0.134	13.0	10.3	109	0.112	43.5	2.6
C_16:0_	154	0.158	15.4	10.3	99	0.102	49.5	2.1
C_18:1_^Δ9^	217	0.223	21.7	10.3	101	0.104	50.5	2.1

a Kinetic constants (V_max_ and *K_m_*) determined using Hanes-Woolf plot.

b nmole of acyl-CoA formed/min/mg of protein.

c s^-1^; determined using MW of FadD1 (61,655) and FadD2 (61,373).

d mM of ATP or FA.

e mM^-1^ s^-1^; represents enzyme catalytic efficiency.

### *fadD* mutants influence virulence behavior of *P. aeruginosa*

**Figure 6:**
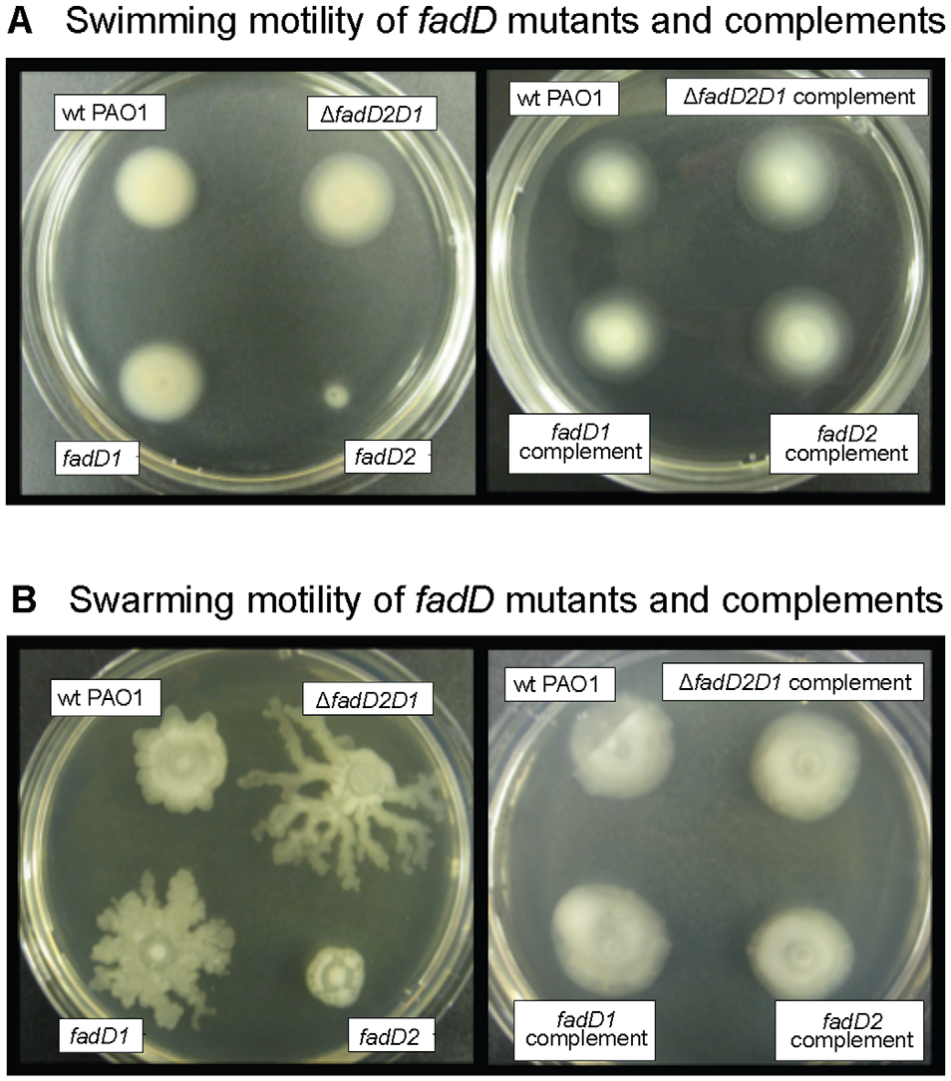
Altered swimming and swarming motility of *P. aeruginosa fadD* mutants. (A) Swimming motility of *fadD* mutants and their complements. (B) Swarming migration of *fadD* mutants and their complements. These figures are representative of multiple experiments. Strain designation is the same as shown in Table 3: wildtype PAO1, P007; *fadD1* ^-^, P175; *fadD2* ^-^, P547; Δ*fadD2D1*, P177; *fadD1* ^-^ complement, P541; *fadD2* ^-^ complement, P549; and Δ*fadD2D1* complement, P543.

Based on work in other bacteria that showed an interconnection between *fadD* genes and expression of virulence factors [37]–[43], we sought to determine if a similar connection existed in *P. aeruginosa*. Increased swarming motility of *S. meliloti*, leading to altered virulence, was previously attributed to hyperflagellation observed by transmission electron microscopy (TEM) [43]. While no apparent differences in structure or numbers of flagella were observed for the *fadD* mutants compared to wildtype PAO1 using TEM in the current study (data not shown), we showed that *fadD* mutations could still significantly influence swarming and swimming motility in *P. aeruginosa* (Fig. 6). The *fadD2* mutant was severely defective in swimming and swarming motility relative to the wildtype PAO1 strain (Fig. 6). Although the *fadD1* single mutant showed no apparent difference in swimming motility, it displayed increased swarming migration compared to PAO1. Swarming was most pronounced in the Δ*fadD2D1* mutant. In the Δ*fadD2D1* mutant, it was very interesting to observe that the *fadD1* mutation suppressed the swarming and swimming defects of the *fadD2* mutation. Each complemented strain showed that swimming and swarming motility could be restored to wildtype levels, indicating no unforeseen secondary or polar mutations affected these behaviors.

**Figure 7:**
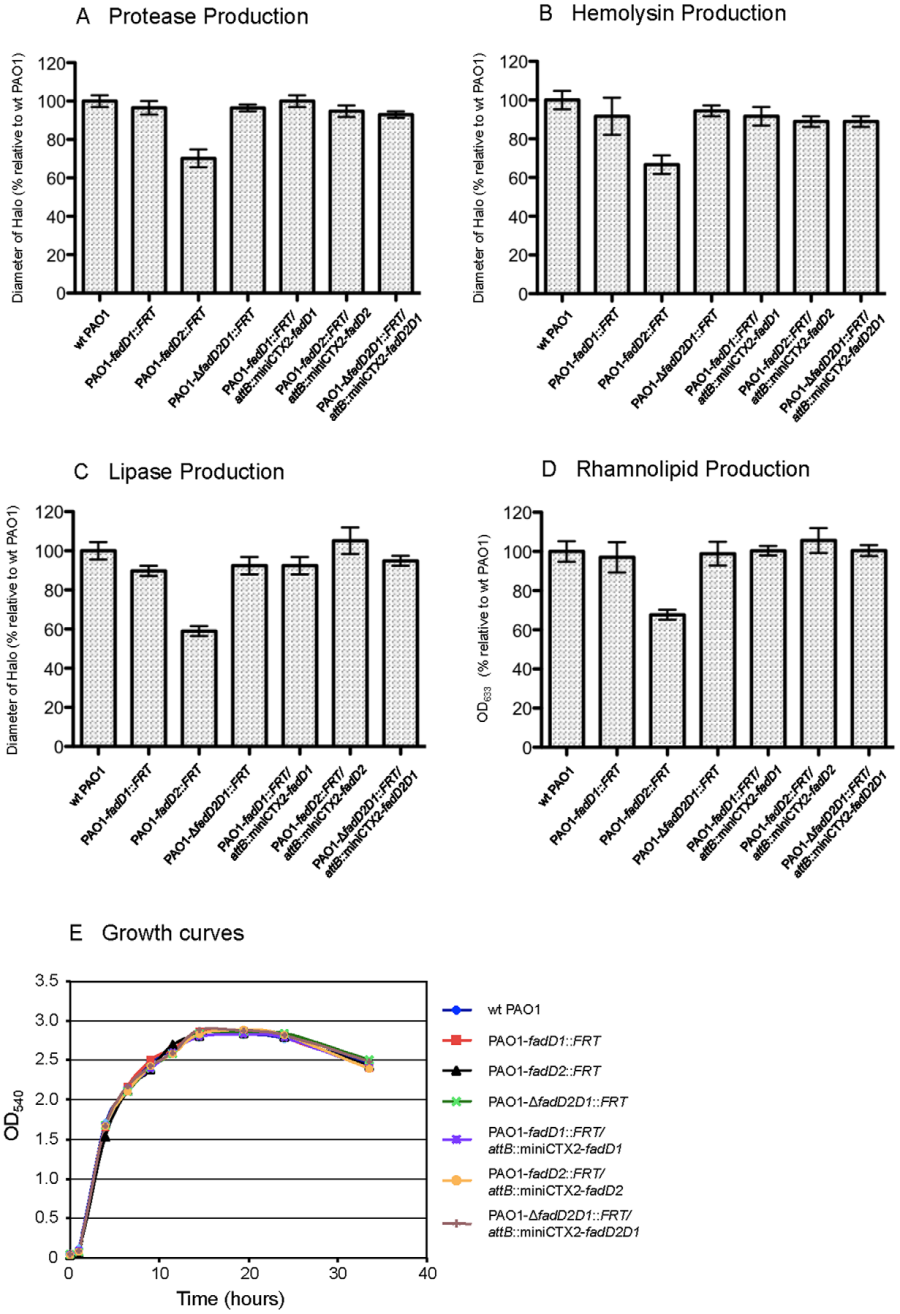
Analysis of protease, hemolysin, lipase, and rhamnolipid production by *P. aeruginosa fadD* mutants. The *fadD2* mutant displayed significantly decreased production of proteases (A), hemolysins (B), lipases (C), and rhamnolipids (D), while no growth defects in LB were observed (E). These assays were conducted in triplicate and are expressed as a percentage of the mean value of the wildtype PAO1 ± s.e.m.

The production of other virulence factors was also monitored for the *fadD* mutants. Interestingly, the *fadD2* mutant showed significantly decreased production of total hemolysins, proteases, lipases, and rhamnolipids (Fig. 7). No other mutant or complement showed noticeable decreases in the production of these virulence factors compared to the wildtype PAO1 strain. The supression of the *fadD2* mutation by the *fadD1* mutation, in the Δ*fadD2D1* mutant, reversed the reduction in virulence factor expression seen in the single *fadD1* mutation alone (Fig. 7). Similar suppression was observed in swimming and swarming motilities (Fig. 6). The altered virulence behaviors and suppression were not due to differences in growth-rates or overall final cell densities, as all seven strains (i.e. wildtype, mutants, and complements) grew identically in LB media prior to testing for these virulence traits (Fig. 7E). Mechanisms governing these differences remain to be elucidated.

### Compromised ability of *fadD* mutants to degrade FA and PC leads to reduced *P. aeruginosa* fitness in mice

**Figure 8:**
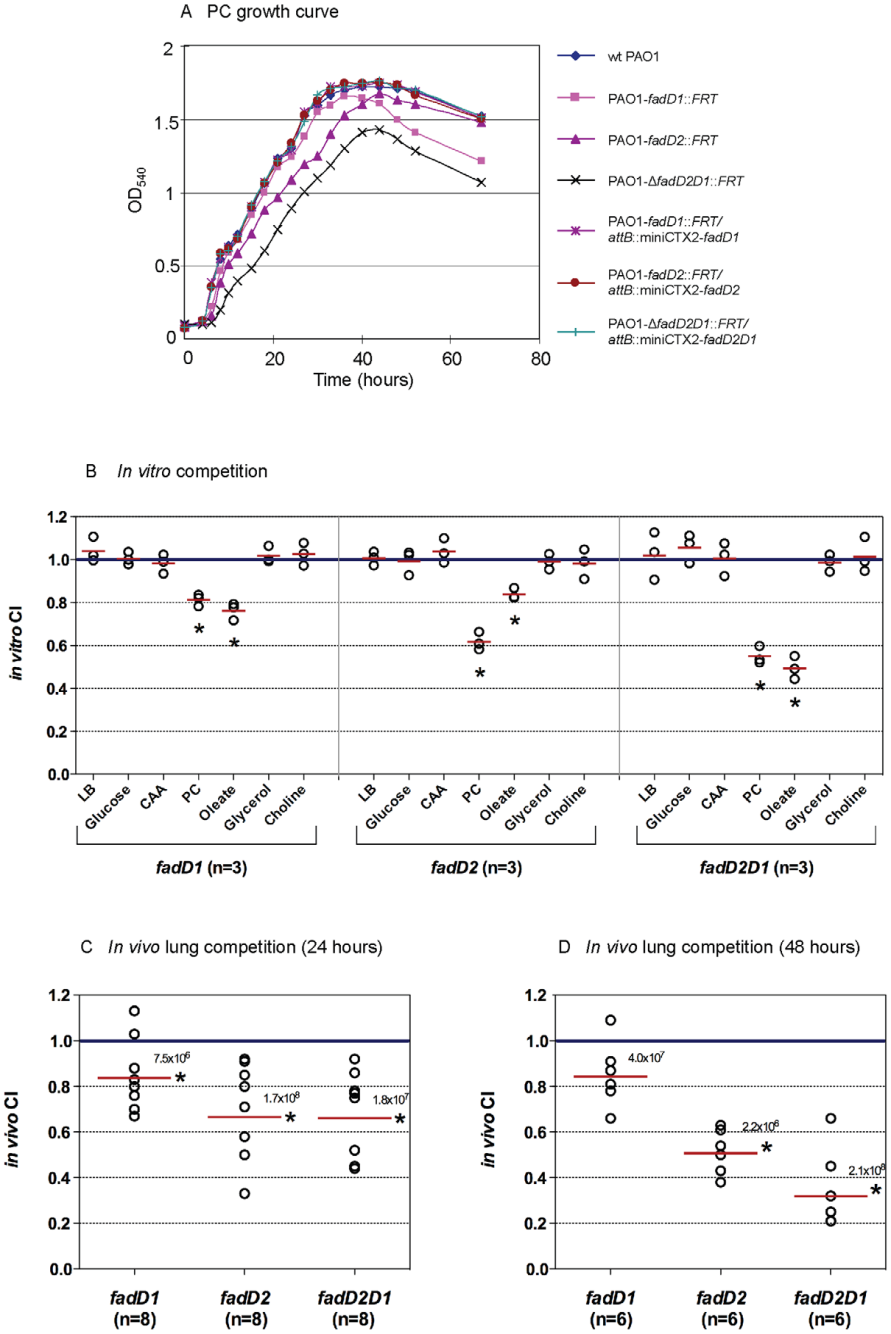
Growth analysis on phosphatidylcholine and competition studies. (A) The Δ*fadD2D1* mutant exhibited a growth defect when grown on PC as a sole carbon source, while the *fadD2* mutant had a delayed log phase compared to the wildtype PAO1 strain. The growth defects were fully recovered in complemented strains, as they had identical growth rates compared to the wildtype PAO1 strain. (B) *In vitro* competition studies of the various *fadD* mutants and their complemented strains in different growth media (*n* = the number of independent *in vitro* competition experiments performed with each carbon source). *In vivo* lung competition of the various *fadD* mutants and their complemented strains after 24 h (C) and 48 h (D). *n* equals the number of mice in each group that were inoculated with a total of 6×10^6^ CFU/mouse. The solid red line indicates the geometric mean of the competitive indices (CI) in each competition group. CI<1 indicates the *fadD* mutant was less competitive than its complemented strain in various growth media (B) or within the lungs (C and D) (*, *P*<0.05 based on one sample *t* test) [47]. Numbers above the red line represent the average total recovered CFU/mouse for each competition group.

Since *fadD1* and *fadD2* are expressed *in vivo* during CF lung infections [12] and are potentially important for PC degradation (Fig. 1A), it was necessary to determine whether these mutants are deficient in growth on PC. Growth analysis on PC showed only slight decreases in the maximum cell density of the individual single *fadD* mutants, while the *fadD2* mutant exhibited a delayed log phase (Fig. 8A). The Δ*fadD2D1* mutant exhibited the greatest growth defect, while the single and double *fadD* complements restored growth to wildtype levels. Since PC is the major component (70%) of the essential lung surfactant [46] and is a potential nutrient source *in vivo* [12], it was important to assess whether the growth defects of these mutants on PC would result in decreased fitness *in vivo*.

*In vitro* competition between the various *fadD* mutants and their complements were first tested to determine if the defect reduced their ability to utilize various sole carbon sources. Each mutant and complement pair was inoculated into seven growth media with different sole carbon sources including LB, CAA, glucose, PC, C_18:1_^Δ9^, choline, and glycerol, and bacterial CFU were determined after 24–48 h growth (Fig. 8B). As, expected, all three single or double *fadD* mutants were less competitive than their corresponding complements but only in media containing PC or C_18:1_^Δ9^ as sole carbon sources. Next, to evaluate the fitness of the *fadD* mutants within the lung environment, *in vivo* competition between the mutants and their complements was analyzed. Following intratrachael inoculation of equal ratios of each mutant and its complement pair into BALB/c mice (6×10^6^ CFU/animal), bacterial CFU recovered from the lungs were determined 24 h and 48 h postinfection and the competitive index (CI) was calculated (Fig. 8C and 8D). The CI is defined as the ratio of mutant CFU relative to CFU of the respective complement [47]. In all of these competition experiments, with the exception of the *fadD2* mutant after 48 h, the average total CFU/mouse recovered was greater than the initial inoculum showing that these strains maintained the ability to replicate within the mouse lung. Although the *fadD1* single mutant showed decreased competitive fitness within the lung compared to the complement, the CI of the *fadD1* mutant remained relatively unchanged between the two time points analyzed. At 24 h postinfection, all of the mutants exhibited decreased competition levels relative to their respective complements and the *fadD2* and Δ*fadD2D1* mutant strains showed greater reduced fitness than the *fadD1* mutant. By allowing the infections to persist for 48 h, the reduced CI of the *fadD2* mutant showed a significantly higher defect in competitive fitness. By 48 h, the CI of the Δ*fadD2D1* mutant was half of that observed at 24 h. Clearly, the Δ*fadD2D1* mutant with significantly reduced ability to degrade PC (Fig. 8A), while showing no altered virulence factor secretion (Fig. 7), had its competitive fitness reduced by three-fold. This strongly suggests that the ability to degrade PC as a nutrient source allows *P. aeruginosa* to replicate within the lung environment.

## Discussion

This study focused on characterizing two *P. aeruginosa* acyl-CoA synthetases (FadD1 and FadD2), which are expressed during lung infection in CF patients suggesting their importance in lipid degradation for bacterial replication [12]. The transcriptional profile and substrate preferences of FadD1 and FadD2 were determined to initially shed light onto why *P. aeruginosa* has this genetic redundancy. The *fadD1* and *fadD2* of *P. aeruginosa* were differentially regulated in response to the type of available FA (Fig. 3), suggesting that each FadD has a different FA substrate preference. While both *fadD1* and *fadD2* were controlled by their common and independent promoters, the expression of *fadD1* downstream could be partially attenuated by some FAs due to a putative intergenic Rho-independent transcriptional-terminator (Fig. 2). Based on the growth defects, both *fadD* genes are individually important for the degradation of all chain-length FAs tested. These results are further supported by the observation that the Δ*fadD2D1* mutant had the greatest growth defect on all chain-length FAs (Fig. 4). These growth defects may be due to a bottleneck in the conversion of exogenous FA to acyl-CoA created by the inactivation of two genes that facilitate this process. The complementation study in *E. coli* was initially inconclusive with respect to metabolism of SCFA and some MCFA substrates by *P. aeruginosa* FadDs (Table 1), as other *E. coli* Fad-proteins (e.g. FadE, FadA and FadB) do not allow the metabolism of shorter-chain FAs [45]. However, the kinetic parameters, especially the catalytic efficiencies (Table 2), provided more precise biochemical evidence for the differences in substrate preferences. By comparing the kinetic measurements of these enzymes, it appears that FadD1 preferred LCFA for degradation, while FadD2 was more suited to the degradation of SCFAs and MCFAs (Fig. 5 and Table 2). Together, the enzyme kinetics, gene-fusion, and growth analyses data all support the importance of FadD2 for degradation of SCFA and MCFA and FadD1 is more suited for LCFA degradation (Fig. 3, 4 and 5). Therefore, we can conclude that these proteins have different substrate preferences and are not functionally equivalent.

We showed here that mutations in *fadD* genes, important for FA β-oxidation in *P. aeruginosa*, also influenced two modes of motility and virulence factor expression. Our *fadD2* mutant had reduced swimming and swarming motilities and decreased virulence factor expression (proteases, phospholipases, rhamnolipids, and lipases), while the *fadD1* mutant and the Δ*fadD2D1* double mutant only showed an increased swarming phenotype. In the double mutant background, the presence of the *fadD1* mutation suppresses the phenotype of the *fadD2* mutation (Figs. 6 and 7). It will be interesting to determine the exact cause of the phenotypic suppression in future investigations. At this point, we speculate that the reduced swarming phenotype of the *fadD2* mutant is due to decreased production of rhamnolipids (Fig. 6 and 7), as rhamnolipids were previously shown to be necessary for *P. aeruginosa* swarming motility [48]. These phenotypic differences in virulence factor expression further support the observation that FadD1 and FadD2 are not functionally equivalent. Although we did not exhaust the large list of virulence determinants, nor were able to show the exact method by which FadD2 influences their expression, this characterization of several virulence factors links Fad and virulence factor expression in *P. aeruginosa*.

Additionally, the expression of genes *in vivo* that encode proteins with β-oxidative activity, along with several other PC degradation genes, strongly support the hypothesis that lipids within the lung may be important nutrient sources for *P. aeruginosa* [12] and serve as signals to control virulence factor expression [13]. Phospholipase- and lipase-derived components of PC (LCFA, glycerol, and phosphorylcholine) could individually serve as sole carbon sources (Fig. 1A) and provide nitrogen and phosphorous contributing to virulence [49], [50]. Of these three PC components, the two LCFAs from each PC molecule yield the most carbon and energy. Therefore, the determination that both FadD1 and FadD2 were important for LCFA degradation was pivotal as PC, the major component of lung surfactant, is primarily composed of LCFA (C_16:0_, 50–60%; C_14:0_, C_16:1_, C_18:1_, and C_18:2_ each at 10–20%) [46]. To that end, we analyzed the growth of these mutants on PC as a sole carbon source. The delayed log-phase of the *fadD2* mutant is likely due to the decreased expression of lipase and phospholipase, thereby reducing the cleavage rate of exogenous PC into its usable components and thus slowing growth. Since it was shown that the Δ*fadD1D2* mutant had no apparent deficiencies in lipase or phospholipase expression, yet exhibited the greatest decrease in growth on all FAs tested, we believe that its reduced growth on PC is attributed to a reduced ability to degrade FAs, as this double mutant degrades phosphorylcholine and glycerol as well as the complement. Since the *fadD* mutants fully retained the ability to degrade choline and glycerol and only had reduced levels of FA degradation, it was not surprising that the Δ*fadD1D2* double mutant could still degrade PC.

Because PC is a major lung surfactant common in all mammals, including mice [51], a mouse lung infection model [52] was utilized to analyze the competitive growth *in vivo* between the *P. aeruginosa fadD* mutants and their complements. Replication of the *fadD2* mutant was observed after 24 h, while all other strains showed an increase after 48 h, indicating all strains were able to replicate *in vivo*. This could be explained by the fact that our Δ*fadD1D2* double mutant still had significant ability to degrade PC and its components, and that *P. aeruginosa* expresses genes in the lung for both amino acid and PC degradation [12] and possibly DNA [53]. After 24 h and 48 h, all mutants showed lower *in vivo* fitness than their complements, which means that lipids are significant nutrient sources in the mouse lung. The CIs of the *fadD2* mutant were consistently lower than those of the *fadD1* mutant after both 24 h and 48 h, attributed to the *fadD2* mutant's decreased production of virulence factors. Although the Δ*fadD2D1* mutant only showed partial defect in PC degradation and no effect in virulence factor expression or the ability to grow with amino acids, this partial defect in PC degradation translated into significantly reduced *in vivo* lung fitness. This is further supported by the *in vitro* competition results where all single and double *fadD* mutants exhibited competition defect only on PC and C_18:1_^Δ9^. Therefore, the significantly lowered CI after 48 h for the double Δ*fadD2D1* mutant can only be due to a reduced ability to degrade PC as a nutrient source for replication in the mouse lung.

In this study, a pathophysiological link between the acquisition of lipid nutrients and virulence *in vivo* was established by i) characterizing these two *P. aeruginosa fadD* genes expressed during lung infection in CF patients, ii) determining that there may be some connection between *fad*-genes and the expression of certain virulence traits, and iii) showing that mutations in these genes correspond to a deficiency in the ability to replicate in mouse lungs. These data support results from a previous *in vivo* gene expression study showing that *P. aeruginosa* expresses *glp*-, *bet*-, and especially, *fad*-genes to degrade PC as one of the nutrient sources in the lungs [12]. We predict that *P. aeruginosa* mutants, completely blocked in PC utilization, will have significantly lower *in vivo* fitness, further supporting PC as a significant nutrient source for this important pathogen in mammalian lungs.

## Materials and Methods

### Ethics statement

All animal experiments were conducted in compliance with the NIH (National Institutes of Health) Guide for the Care and Use of Laboratory Animals and were approved by the University of Hawaii Institutional Animal Care and Use Committee (protocol No. 06-023-04).

### Bacterial strains and growth media

Strains and plasmids used in this study are shown in Tables 3 and 4. *E. coli* EPMax10B was routinely used as a strain for cloning and was cultured in Luria-Bertani (LB) medium (Difco). *P. aeruginosa* strain PAO1 and derivatives were cultured on Pseudomonas Isolation Agar or Broth (PIA or PIB; Difco) or LB medium. PAO1-*fadD1*::*FRT*, PAO1-*fadD2*::*FRT*, PAO1-Δ*fadD2D1*::*FRT*, and their complements were cultured in 1x M9 minimal medium +1% Brij-58 (Sigma) +1% casamino acids (CAA) or 0.2% (w/v) of the individual FA, C_4:0_ to C_16:0_, or C_18:1_^Δ9^ (Sigma; Fig. 4), and 1x M9 minimal medium +1% Brij-58 +0.2% phosphatidylcholine (PC, Sigma; Fig. 8A) for growth analyses. The fusion strains PAO1-*fadD1*::*FRT-lacZ/attB*::miniCTX2*-fadD1* and PAO1-*fadD2*::*FRT-lacZ/attB*::miniCTX2*-fadD2* were cultured in 1x M9 +1% Brij-58 +1% CAA±0.1% (w/v) C_4:0_ to C_16:0_, or C_18:1_^Δ9^ for induction studies (Fig. 3C and 3D). For *in vitro* competition studies (Fig. 8B), each mutant/complement mixture of equal cell density was grown in seven different media, including LB, 1x M9 +20 mM glucose, 1x M9 +1% CAA, 1x M9 +1% Brij-58 +0.2% PC, 1x M9 +1% Brij-58 +0.2% C_18:1_^Δ9^, 1x M9 +40 mM glycerol, and 1x M9 +30 mM choline. Stock solutions of all FAs were made as previously described [30]. Unless indicated otherwise, all liquid cultures were grown at 37°C with a shaking speed of 200 r.p.m.

**Table 3:** Bacterial strains used in this study^a^.

Strains	Lab ID^b^	Relevant properties	Reference
***E. coli***			
EPMax10B	E1231	F^-^ λ*^-^ mcrA* Δ*(mrr-hsdRMS-mcrBC) ϕ80dlacZ* Δ*M15* Δ*lacX74 deoR recA1 endA1 araD139* Δ*(ara, leu)7697 galU galK rpsL nupG*	BioRad
K12	E0577	Prototroph	ATCC #23740
K27	E0410	*fadD^-^* (*oldD88*) mutant	[16]
K27-T7	E1063	Gm^r^; K27 with T7 expression system*fadD^-^* (*oldD88*) λ*attB*::T7(*pol-lysS*)-*lacI^q^*-Gm^r^	Lab strain
JWC285	E1560	Km^r^; *fadA*::*Tn10 yfcYX*::*cat fadR*::Km^r^	[44]
K27-*fadR^-^*	E2011	Km^r^; *fadD^-^ fadR*::Km^r^ (transduced from strain JWC285 P1-lysate into strain K27)	This study
***P. aeruginosa***			
PAO1	P007	prototroph	[68]
PAO1-*fadD1*::*FRT*	P175	PAO1 with *fadD1* insertional mutation	This study
PAO1-*fadD2*::*FRT*	P547	PAO1 with *fadD2* insertional mutation	This study
PAO1- Δ*fadD2D1*::*FRT*	P177	PAO1 with *fadD2D1* deletional mutation	This study
PAO1-*fadD1*::*FRT*/*attB*::miniCTX2-*fadD1*	P541	Tet^r^; PAO1-*fadD1*::*FRT* complemented with miniCTX2-*fadD1*	This study
PAO1-*fadD2*::*FRT*/*attB*::miniCTX2-*fadD2*	P549	Tet^r^; PAO1-*fadD2*::*FRT* complemented with miniCTX2-*fadD2*	This study
PAO1-Δ*fadD2D1*::*FRT*/*attB*::miniCTX2-*fadD2D1*	P543	Tet^r^; PAO1-Δ*fadD2D1*::*FRT* complemented with miniCTX2-*fadD2D1*	This study
PAO1-*fadD1*::*FRT-lacZ/* *attB*::miniCTX2-*fadD1*	P518	Tet^r^, Gm^r^; *fadD1* complement strain with *fadD1*-*FRT*-*lacZ* fusion	This study
PAO1-*fadD2*::*FRT-lacZ/* *attB*::miniCTX2-*fadD2*	P520	Tet^r^, Gm^r^; *fadD2* complement strain with *fadD2*-*FRT*-*lacZ* fusion	This study
PAO1-*fadD1*::*FRT/* *mucA*::pUC18	P663	Cb^r^; *fadD1* mutant with pUC18 inserted in *mucA* gene	This study
PAO1-*fadD2*::*FRT/* *mucA*::pUC18	P667	Cb^r^; *fadD2* mutant with pUC18 inserted in *mucA* gene	This study
PAO1-Δ*fadD2D1*::*FRT/* *mucA*::pUC18	P665	Cb^r^; *fadD2D1* mutant with pUC18 inserted in *mucA* gene	This study
PAO1-*fadD1*::*FRT*/*attB*::miniCTX2-*fadD1/* *mucA*::pUC18	P657	Cb^r^, Tet^r^; *fadD1* mutant with pUC18 inserted in *mucA* gene	This study
PAO1-*fadD2*::*FRT*/*attB*::miniCTX2-*fadD2/* *mucA*::pUC18	P659	Cb^r^, Tet^r^; *fadD2* mutant with pUC18 inserted in *mucA* gene	This study
PAO1-Δ*fadD2D1*::*FRT*/*attB*::miniCTX2-*fadD2D1/* *mucA*::pUC18	P661	Cb^r^, Tet^r^; *fadD2D1* mutant with pUC18 inserted in *mucA* gene	This study

a For strains constructed in this study, please see text for further details.

b Please use lab ID for requesting strains.

**Table 4:** Plasmids used in this study^a^.

Plasmids	Lab ID^b^	Relevant properties	Reference
pEX18T	E0055	Ap^r^; gene replacement vector	[55]
pEX18T-*fadD2D1*	E0551	Ap^r^; pEX18T with *fadD2D1*	This study
pEX18T-*fadD1*::Gm-*FRT*	E0635	Ap^r^, Gm^r^; Gm^r^-*FRT*-cassette inserted into *fadD1*	This study
pEX18T-*fadD2*::Gm-*FRT*	E2163	Ap^r^, Gm^r^; Gm^r^-*FRT*-cassette inserted into *fadD2*	This study
pEX18T-Δ*fadD2D1*::Gm-*FRT*	E0455	Ap^r^, Gm^r^; Gm^r^-*FRT*-cassette inserted into *fadD2D1*	This study
pPS856	E0050	Ap^r^, Gm^r^; plasmid with Gm^r^-*FRT*-cassette	[55]
pET15b	E0047	Ap^r^; T7 expression vector	Novagen
pET15b-*fadD1*	E0753	Ap^r^; pET15b with *fadD1*	This study
pET15b-*fadD2*	E0756	Ap^r^; pET15b with *fadD2*	This study
pET15b-*fadD_Ec_*	E2177	Ap^r^; pET15b with *E. coli fadD*	This study
pET15b-*fadD2*::Gm-*FRT*	E2155	Ap^r^, Gm^r^; Gm^r^-*FRT*-cassette inserted into *fadD2*	This study
miniCTX2	E0076	Tet^r^; site-specific integration vector	[56]
miniCTX2-*fadD1*	E2157	Tet^r^; miniCTX2 with cloned *fadD1*	This study
miniCTX2-*fadD2*	E2159	Tet^r^; miniCTX2 with cloned *fadD2*	This study
miniCTX2-*fadD2D1*	E2143	Tet^r^; miniCTX2 with cloned *fadD2D1*	This study
pUC18	E0135	Ap^r^; cloning vector	[69]
pUC18-‘*mucA*’	E1907	Ap^r^; *mucA* internal region cloned into pUC18	This study
pUC19	E0014	Ap^r^; cloning vector	[69]
pUC19-*fadD2D1*	E0545	Ap^r^; pUC19 with cloned *fadD1* and *fadD2*	This study
pUC19-Δ*fadD2D1*::Gm-*FRT*	E0416	Ap^r^, Gm^r^; pUC19-*fadD2D1* with a Gm^r^-*FRT*-cassette insertion	This study
pFRT1-*lacZ*-Gm	E0790	Gm^r^; *FRT*-*lacZ* fusion vector	[29]
pCD13SK-*flp*-*oriT*	E0783	Sp^r^; suicidal Flp-containing plasmid	[29]
pFLP2	E0067	Ap^r^; Flp-containing plasmid	[55]

a For plasmids constructed in this study, please see text for further details.

b Please use lab ID for requesting plasmids.

### General molecular methods

All molecular methods and their components were employed as previously described [54]. Oligonucleotides utilized in this study were ordered through Integrated DNA Technology (IDT, Table 5).

**Table 5:** Primers used in this study.

Number and Name	Sequence^a^
***fadD*** ** cloning**	
302; *fadD*-up-Hind	5′-ATCGGAAGCTTCCGGGTGCTGCTGGCGGAT-3′
303; *fadD*-down	5′-TTCGTGGAGCTGCCGGCGCAAGC-3′
339; *fadD2*-NdeI	5′-CAAGAACATATGCAACCTGAATTCTGGAACG-3′
340; *fadD2*-BamHI	5′-CGGCAAGGATCCGTTTCAGAGCCTTGTAAGGCT-3′
341; *fadD1*-NdeI	5′-TGGGCTCATATGATCGAAAACTTCTGGAAGG-3′
342; *fadD1*-BamHI	5′-GGGGCGGATCCAGGCAACGGCGGACTTACTTC-3′
501; Gm-up-reverse	5′-CATACGCTACTTGCATTACAG-3′
713; *lacZα*	5′-TGTTGGGAAGGGCGATC-3′
1092; *EcfadD*-up-AseI	5′-AAGGATTAATAAGAAGGTTTGGCTTAAC-3′
1093; *EcfadD*-down-BamHI	5′-AACGGGATCCTCAGGCTTTATTGTC-3′
**Promoter mapping**	
372 – *fadD2*-race3^b^	5′-GAGCGCTCGAAGACCTCGA-3′
373 – *fadD2*-race2	5′-GAAGTCGAGACTGTCGGGTA-3′
374 – *fadD2*-race1	5′-TCGTTCCAGAATTCAGGTTG-3′
375 – *fadD1*-race3^b^	5′-ACAGGATGTTCGGGTACTG-3′
376 – *fadD1*-race2	5′-CGGGATTGATCTCGGCAGCA-3′
377 – *fadD1*-race1	5′-GTACTTGTCCTTCCAGAAGT-3′
797 – SMART-IIA^b^	5′-AAGCAGTGGTATCAACGCAGAGTACGCGGG-3′
798 – SMART-IIB	5′-AAGCAGTGGTATCAACGCAGAGT-3′
***mucA*** ** cloning**	
937 – *mucA*-up	5′-GAAGCGGATGAACTCGAG-3′
938 – *mucA*-down	5′-ACTGACGGCGGATTGTTG-3′

a Restriction enzyme sites utilized in this study are underlined.

b Primers synthesized RNase free and HPLC purified.

### Complementation of *E. coli fadD* mutant

The *E. coli fadD^-^ fadR*::Km^r^ mutant (E2011) was engineered by transferring the *fadR*::Km^r^ mutation from JWC285 into the K27 (*fadD^-^*) strain via P1 transduction. The resulting double mutant strain was then used for the complementation study. To construct the *E. coli* complementation vectors, coding regions of *P. aeruginosa fadD1* and *fadD2* were amplified from PAO1 chromosomal DNA using oligos #341 +#342 and #339 + #340, respectively. PCR products were then digested with NdeI + BamHI and ligated individually into pET15b, digested with the same enzymes, yielding pET15b-*fadD1* and pET15b-*fadD2*. As a control for the complementation study, the *E. coli fadD* gene (*fadD_Ec_*) was also amplified from strain K12 chromosomal DNA using oligos #1092 + #1093. The 1.8-kb PCR product was digested with AseI + BamHI, and ligated with pET15b digested with NdeI + BamHI, yielding pET15b-*fadD_Ec_*. These three vectors, pET15b-*fadD1*, pET15b-*fadD2*, and pET15b-*fadD_Ec_*, were introduced into *E. coli* strain E2011 for complementation. E2011, harboring each complementation vector, was patched on 1x M9 minimal medium + 1% Brij-58 +1% CAA or 0.2% various fatty acids, and growth was determined after 3 days incubation at 37°C (Table 1).

### Construction of PAO1 *fadD* mutant and complementation strains

Three *fadD* mutant strains (PAO1-*fadD1*::*FRT*, PAO1-*fadD2*::*FRT*, and PAO1-Δ*fadD2D1*::*FRT*) were engineered, respectively, using three allelic-replacement plasmids (pEX18T-*fadD1*::Gm-*FRT*, pEX18T-*fadD2*::Gm-*FRT*, and pEX18T-Δ*fadD2D1*::Gm-*FRT*) as previously described [55]. These gene-replacement vectors were constructed by inserting the SmaI Gm^r^-*FRT* cassette at the EcoRV site to inactivate *fadD1* gene, the SmaI site to inactivate *fadD2* gene, or at the deleted *fadD2D1* SmaI-EcoRV locus. These PAO1 *fadD* mutants were confirmed by PCR (data not shown).

These newly engineered mutant strains, PAO1-*fadD1*::*FRT*, PAO1-*fadD2*::*FRT*, and PAO1-Δ*fadD2D1*::*FRT*, were complemented using the relevant gene(s) on the miniCTX2 single copy integration vector as described previously [56]. The resulting strains, PAO1-*fadD1*::*FRT*/*attB*::miniCTX2-*fadD1*, PAO1-*fadD2*::*FRT*/*attB*::miniCTX2-*fadD2*, and PAO1-Δ*fadD2D1*::*FRT*/*attB*::miniCTX2-*fadD2D1*, were used in the growth curve experiments (Fig. 4 and 8A). Controls were also performed with the empty miniCTX2 integrated into each mutant strain.

### Growth characterization of *fadD* mutant and complemented strains

The *fadD* mutants, their corresponding complemented strains, and the PAO1 wildtype strain were initially grown overnight in PIB medium. The overnight cultures were centrifuged and the cell pellets were washed twice with 1x M9 minimal media and resuspended with equal volumes of the same 1x M9 media. The cell resuspensions were then diluted 100-fold in 1x M9 +1% Brij-58 +1% CAA or 0.2% of the individual FAs (C_4:0_ to C_16:0_, or C_18:1_^Δ9^; Fig. 4) or 0.2% PC (Fig. 8A), and growth was then initiated. At each time point, aliquots of individual cultures were diluted 4-fold in 4% Brij-58 (pre-incubated at 42°C) to clarify any insoluble FA and OD_540_ measurements were taken.

### Construction of *fadD1-lacZ* and *fadD2-lacZ* fusion strains and induction by FAs

To take advantage of the native *fadD1*-promoter and create a transcriptional fusion of P*_fadD1_-lacZ*, pFRT1-*lacZ* was integrated at the *FRT* locus in strain PAO1-*fadD1*::*FRT* as previously described [29]. The resulting fusion strain, PAO1-*fadD1*::*FRT-lacZ*, was PCR confirmed using oligos #341 + #713, which are specific for the *fadD1* and *lacZ* genes, respectively. Similarly, PAO1-*fadD2*::*FRT-lacZ* was constructed and PCR confirmed using oligos #377 + #501 (data not shown). Complementation vectors miniCTX2-*fadD1* and miniCTX2-*fadD2* were then integrated into these newly developed *lacZ*-fusion strains, to yield two complemented fusion strains, PAO1-*fadD1*::*FRT-lacZ/attB*::miniCTX2-*fadD1* and PAO1-*fadD2*::*FRT-lacZ/attB*::miniCTX2-*fadD2*, respectively.

β-Galactosidase activities were measured for these two complemented fusion strains under various growth conditions. Cells were first grown overnight in PIB medium, washed twice with one volume of 1x M9, and resuspended in an equal volume of the same medium. Cell resuspensions were then diluted 100-fold into fresh 1x M9 +1% Brij-58 +1% CAA±0.1% of the individual FAs (C_4:0_ to C_16:0_, or C_18:1_^Δ9^), and growth curve experiments were performed. Cell cultures were taken at mid-log phase (OD_540_ ∼2.0) and β-galactosidase assays were performed in triplicate and Miller Units (mean±s.e.m.) were determined [57] (Fig. 3).

### FadD1 and FadD2 purification

Histidine-tagged FadD1 and FadD2 were expressed on the pET15b vector and purified using a Ni^+^-NTA column (Qiagen, Valencia, CA) as described elsewhere [58]. The *E. coli* K27-T7 (*fadD* ^-^) strain was used for protein expression to prevent any possible *E. coli* FadD contamination in protein preparations (Fig. 5A).

### Measurement of fatty acyl-CoA synthetase (FadD1 and FadD2) activity

Fatty acyl-CoA synthetase activity was monitored using Ellman's reagent, as previously described in several studies, to detect the amount of free thiol (i.e. CoASH used in the reaction) [23], [59]–[61]. Reactions (450 µl total) were prepared with 20 µg of purified FadD1 (or FadD2) in a reaction buffer containing final concentrations of 150 mM Tris-HCl (pH 7.2), 10 mM MgCl_2_, 2 mM EDTA, 0.1% Triton X-100, 5 mM ATP, 0.5 mM coenzyme A (CoASH), and an individual FA (30 to 300 µM) in thin-walled glass tubes. Briefly, to perform the reaction, each mixture was assembled containing all components above (excluding CoASH) and the 405 µl mixture was pre-incubated at 37°C for 3 min. The reaction was then initiated with the addition of 45 µl of CoASH (5 mM stock in 20 mM Tris-HCl, pH 6.7, diluted to a final concentration of 0.5 mM) that was pre-incubated at 37°C for 3 min, quickly mixed, and incubated at 37°C during the course of the reaction. Immediately after mixing, a time zero point was taken by removing 75 µl from the 450 µl reaction mix and adding it to 600 µl of 0.4 mM 5,5′-dithiobis(2-nitrobenzoic acid) (DTNB, dissolved in 0.1 M potassium phosphate at pH 8.0) and the A_412_ were measured. Subsequent 75 µl aliquots of the reaction were taken at 20-sec intervals and mixed with DTNB for additional measurements. Additionally, control experiments without FadD enzymes were performed exactly as above to show no change in absorbance at 412 nm and verify the stability of CoASH and DTNB under these conditions. Reactions with FadD were repeated to obtain triplicate data for each FA at each concentration. For each FA substrate, decreases in A_412_ values (loss of CoASH) over time were used to calculate the initial velocity (*V_0_*) for each FA concentration (Fig. 5B and 5C). The maximum velocity (*V*_max_) of the enzymes and affinity for the different substrates (Michaelis constant, *K_m_*) were then determined using the Hanes-Woolf plot, rather than the Lineweaver-Burk plot, for increased accuracy [62]. To determine the *V*_max_ and *K_m_* for the substrate ATP, the same procedure was followed, except that the concentration of C_18:1_^Δ9^ was constant (1 mM) and varying concentrations of ATP (0.05 to 2.5 mM) were used (Table 2).

### Motility assays

Strains for swarming and swimming were grown overnight in LB medium. Cell pellets of 500 µl culture aliquots were washed twice with equivalent volumes of 1x M9 medium and resuspended in equal volumes of the same medium. Swarming motility was assayed by spotting 5 µl of the resuspended cultures onto BM2-glucose swarm agar plates, made as described previously [63]. Swimming motility was assayed by pin-stabbing 0.3% LB agar plates with the overnight liquid cultures grown in LB. All inoculated plates were allowed to dry at room temperature for 10 min, incubated at 37°C for 16 to 18 h, and motility zones were compared (Fig. 6).

### Protease, phospholipase, lipase, and rhamnolipid detection

Strains were grown in LB medium and cultures were used for OD_540_ measurements at various time points (Fig. 7E). For protease, phospholipase, and lipase assays, clarified supernatant was obtained at 24 h from 1 ml culture aliquots centrifuged (16,000× *g*) for 2 min at 4°C and filtered through 0.2 µm hydrophilic PVDF filters (Fisher Scientific). To quantify protease and phospholipase activities, 4-mm diameter holes were punched into 2% skim milk NB agar protease plates or blood agar phospholipase plates (PML Microbiological) and filled twice with 50 µl of each cell-free supernatant, respectively. Both the skim milk and blood agar plates were incubated at 37°C for 18 h before analyzing. Similarly, 50 µl of the same cell-free supernatants were applied five times into 4-mm holes in rhodamine B agar plates [64], and the plates were imaged using a UV transilluminator after incubation at 37°C for 3 days to visualize lipase activity. These plate-based assays were conducted in triplicate and the clearance zone diameters for skim milk and blood agar plates or the fluorescent halo diameters for the rhodamine B plates were measured and compared by percentage conversion relative to the wildtype PAO1 value and were expressed as an average ± s.e.m (Fig. 7A, 7B and 7C).

Rhamnolipid production was assessed using a previously published methylene blue complexation assay [65]. All strains were grown for 24 h in LB medium and 1.5 ml of clarified supernatant for rhamnolipid extraction was obtained from each culture by room temperature centrifugation (16,000× *g*). This assay was conducted in triplicate and average absorbance was compared by percentage conversion relative to the wildtype PAO1 value and was expressed as an average ± s.e.m (Fig. 7D).

### Promoter mapping

The transcriptional start sites of the *fadD1* and *fadD2* genes were determined as previously described [30]. Briefly, PAO1 was grown in 1x M9 minimal media supplemented with 0.2% C_16:0_ to mid-log phase. This FA was chosen prior to the gene induction studies and, in retrospect, it was as appropriate as any other FA to map the *fadD* promoters. Cells were harvested and total RNA was isolated to perform cDNA synthesis using a SMART-IIA primer (#797, Table 5) and the first gene-specific primer (#375 for *fadD1* and #372 for *fadD2*, Table 5). The cDNA was subsequently used as the template in PCR, using oligos #798 + #376 and #798 + #373 for *fadD1* and *fadD2*, respectively. Finally, the PCR product was sequenced using a second nested oligo #377 for *fadD1*, or #374 for *fadD2* (Fig. 2).

### Northern blot analysis

Wildtype strain, PAO1, was grown in 1x M9 minimal medium supplemented with 0.2% C_8:0_, C_14:0_, or C_18:1_^Δ9^ as sole carbon sources. After reaching mid-log phase (OD_540_∼1.0), cells were harvested at 4°C and total RNA was isolated. Thirty µg of each RNA sample was used for northern analysis as described previously [66]. The *fadD1* and *fadD2* genes were PCR amplified from pET15b-*fadD1* and pET15b-*fadD2* using oligos #341 + #342, and #339 + #340, respectively, and used individually as probes (Fig. 3A and 3B).

### *In vitro* and *in vivo* competition studies

Various *fadD* mutant strains (*fadD1*, *fadD2* and *fadD2D1* mutants) and their corresponding complemented strains were utilized for the *in vitro and in vivo* competition studies (Table 3). A *mucA* insertional mutation was introduced into all strains to overproduce alginate, as we used a mouse model to allow these mucoid strains to survive and replicate in the lung as described previously [52]. Briefly, a 450-bp internal region of the *mucA* gene was PCR amplified from PAO1 chromosomal DNA using oligos #973 and #974 and cloned into the PvuI site of pUC18. The resultant vector pUC18-‘*mucA*’ was electroporated into the various *fadD* mutant/complemented strains and the mucoid transformants were selected on PIA plates supplemented with 500 µg/ml carbenicillin (Cb500). One mucoid colony of each mutant/complemented strain was then inoculated separately in 3 ml of PIB + Cb500. After 24 h of incubation in a shaking incubator at 37°C, these cultures were diluted 100 times into 5 ml of fresh PIB + Cb500 and grown overnight. Three ml of each overnight culture was centrifuged (20,000× *g*) for 10 min at 4°C and clarified supernatants were collected. The cell density of each culture was calculated by plating 10-fold serial dilutions on LB plates. Each culture was then adjusted to 2×10^8^ CFU/ml in its own clarified supernatant, obtained above. At this point, each diluted *fadD* mutant strain (*fadD1*, *fadD2* and *fadD2D1* mutants) and its corresponding complemented strain were mixed at a 1∶1 CFU ratio and the resulting mixtures (*fadD1*/complement, *fadD2*/complement, and *fadD2D1*/complement) were used for inoculation into various growth media (*in vitro* competition) or mouse lungs (*in vivo* competition).

For *in vitro* competition, each mutant/complement mixture of equal cell density was diluted 100x into various growth media with LB, glucose, CAA, PC, C_18:1_^Δ9^, glycerol, or choline as sole carbon sources. All cultures were grown at 37°C with shaking for 1–2 days until the total cell densities reached ∼1×10^9^ CFU/ml. Bacteria were then quantified by plating dilutions onto LB plates with and without tetracycline to determine the total number of bacteria (growth with no tetracycline) and the number of complemented bacteria (growth with tetracycline). These numbers were used to determine the *in vitro* CI (CFU_mutant_/CFU_complement_ when grown in media) [47]. All experiments were performed in triplicate and statistical analysis was performed using Graphpad Prism 5.0 software (Fig. 8B).

Male BALB/c mice, 6–8 weeks old, were purchased from Charles River Laboratories and used in this *in vivo* competition study [52]. Before challenge, the mice were anesthetized by the intraperitoneal injection of 100 mg/kg ketamine and 10 mg/kg xylazine. Thirty µl of the mutant/complemented strain mixture (3×10^6^ CFU of each) was inoculated intratracheally into BALB/c mice using the BioLITE Intubation System (Braintree Scientific). After 24 or 48 h, mice were humanely euthanized and lungs were harvested and homogenized. Bacteria were quantified by plating dilutions onto growth media with and without tetracycline to determine the total number of bacteria (growth with no tetracycline) and the number of complemented bacteria (growth with tetracycline). These numbers were used to determine the *in vivo* CI (CFU_mutant_/CFU_complement_ when grown in mouse lungs) [47]. A control condition was included using PAO1-*mucA*::pUC18/PAO1-*mucA*::pUC18-miniCTX2 to show that no competitive advantage or disadvantage was conferred by the presence of the Tet^r^ marker during in vivo growth (data not shown). Statistical analysis was performed using Graphpad Prism 5.0 software (Fig. 8C and 8D).

## References

[1] Rahme LG, Stevens EJ, Wolfort SF, Shao J, Tompkins RG (1995). Common virulence factors for bacterial pathogenicity in plants and animals.. Science.

[2] Abd H, Wretlind B, Saeed A, Idsund E, Hultenby K (2008). *Pseudomonas aeruginosa* utilizes its type III secretion system to kill the free-living amoeba *Acanthamoeba castellanii*.. J Eukaryot Microbiol.

[3] Matz C, Moreno AM, Alhede M, Manefield M, Hauser AR (2008). *Pseudomonas aeruginosa* uses type III secretion secretion system to kill biofilm-associated ameobae.. ISME J.

[4] Weir TL, Stull VJ, Badri D, Trunck LA, Schweizer HP (2008). Global gene expression profiles suggest an important role for nutrient acquisition in early pathogenesis in a plant model of *Pseudomonas aeruginosa* infection.. Appl Environ Microbiol.

[5] Baltch AL, Griffin PE (1977). *Pseudomonas aeruginosa* bacteremia: clinical study of 75 patients.. Am J Med Sci.

[6] Bowton DL (1999). Nosocomial pneumonia in the ICU-year 2000 and beyond.. Chest.

[7] Lode H, Raffenberg M, Erbes R, Geerdes-Fenge H, Mauch H (2000). Nosocomial pneumonia: epidemiology, pathogenesis, diagnosis, treatment and prevention.. Curr Opin Infect Dis.

[8] Richards MJ, Edwards JR, Culver DH, Gaynes RP (1999). Nosocomial infections in medical intensive care units in the United States.. Crit Care Med.

[9] Doring G (1997). Cystic fibrosis respiratory infections: interactions between bacteria and host defense.. Monaldi Arch Chest Dis.

[10] Greenberger PA (1997). Immunologic aspects of lung diseases and cystic fibrosis.. J Am Med Assoc.

[11] Stanier RY, Palleroni NJ, Doudoroff M (1966). The aerobic pseudomonads: a taxonomic study.. J Gen Microbiol.

[12] Son MS, Matthews WJJ, Kang Y, Nguyen DT, Hoang TT (2007). *In vivo* evidence of *Pseudomonas aeruginosa* nutrient acquisition and pathogenesis in the lungs of cystic fibrosis patients.. Infect Immun.

[13] Miller RM, Tomaras AP, Barker AP, Voelker DR, Chan ED (2008). *Pseudomonas aeruginosa* twitching motility-mediated chemotaxis toward phospholipids and fatty acids: specificity and metabolic requirements.. J Bacteriol.

[14] Black PN, DiRusso CC (1994). Molecular and biochemical analyses of fatty acid transport, metabolism, and gene regulation in *Escherichia coli*.. Biochim Biophys Acta.

[15] Clark DP, Cronan JE, Neidhardt FC, Curtiss R, Ingraham JL, Lin ECC, Brooks Low K (1996). Two-carbon compounds and fatty acids as carbon sources.. *Escherichia coli* and *Salmonella*: cellular and molecular biology, 2nd ed.

[16] Overath P, Pauli G, Schairer HU (1969). Fatty acid degradation in *Escherichia coli*: An inducible acyl-CoA synthase, the mapping of old-mutations, and the isolation of regulatory mutants.. Eur J Biochem.

[17] Black PN, DiRusso CC, Metzger AK, Heimert TL (1992). Cloning, sequencing, and expression of the *fadD* gene of *Escherichia coli* encoding acyl coenzymeA synthase.. J Biol Chem.

[18] Weimar JD, DiRusso CC, Delio R, Black PN (2002). Functional role of fatty acid acyl-coenzyme A synthetase in the transmembrane movement and activation of exogenous long-chain fatty acids.. J Biol Chem.

[19] Black PN, Zhang Q, Weimar JD, DiRusso CC (1997). Mutational analysis of a fatty acyl-coenzyme A synthetase signature motif identifies seven amino acid residues that modulate fatty acid substrate specificity.. J Biol Chem.

[20] Black PN (1988). The *fadL* gene product of *Escherichia coli* is an outer membrane protein required for uptake of long-chain fatty acids and involved in sensitivity to bacteriophage T2.. J Bacteriol.

[21] Black PN (1991). Primary sequence of the *Escherichia coli fadL* gene encoding an outer membrane protein required for long-chain fatty acid transport.. J Bacteriol.

[22] Kumar GB, Black PN (1993). Bacterial long-chain fatty acid transport. Identification of amino acid residues within the outer membrane protein FadL.. J Biol Chem.

[23] Groot PHE, Scholte HR, Hulsmann WC (1976). Fatty acid activation: specificity, localization, and function.. Adv Lipid Res.

[24] DiRusso CC, Heimert TL, Metzger AK (1992). Characterization of FadR, a global transcription regulator of fatty acid metabolism in *Escherichia coli*.. J Biol Chem.

[25] Raman N, Black PN, DiRusso CC (1997). Characterization of the fatty acid-responsive transcriptional factor FadR.. J Biol Chem.

[26] van Aalten DMF, DiRusso CC, Knudsen J (2001). The structural basis of coenzyme A-dependent regulation of the transcription factor FadR.. EMBO J.

[27] Campbell JW, Cronan JE (2002). The enigmatic *Escherichia coli fadE* gene is *yafH.*. J Bacteriol.

[28] Kazakov AE, Rodionov DA, Alm E, Arkin AP, Dubchak I (2009). Comparative genomics of regulation of fatty acid and branched-chain amino acid utilization in proteobacteria.. J Bacteriol.

[29] Son MS, Nguyen DT, Kang Y, Hoang TT (2008). Engineering of *FRT-lacZ* constructs: induction of the *Pseudomonas aeruginosa fadBA1* operon by medium and long-chain fatty acids.. Plasmid.

[30] Kang Y, Nguyen DT, Son MS, Hoang TT (2008). The *Pseudomonas aeruginosa* PsrA responds to long-chain fatty acid signals to regulate the *fadBA5* β-oxidation operon.. Microbiology.

[31] Imamura S, Ueda S, Mizugaki M, Kawaguchi K (1990). Purification of the multienzyme complex for fatty acid oxidation from *Pseudomonas fragi* and reconstitution of the fatty acid oxidation system.. J Biochem.

[32] Sato S, Hayashi M, Imamura S, Ozeki Y, Kawaguchi A (1992). Primary structure of the genes, *faoA* and *faoB*, from *Pseudomonas fragi* B-0771 which encode the two subunits of the HDT multienzyme complex involved in fatty acid β-oxidation.. J Biochem.

[33] Sato S, Imamura S, Ozeki Y, Hayashi M, Kawaguchi A (1992). Induction of enzymes involved in fatty acid β-oxidation in *Pseudomonas fragi* B-0771 cells grown in media supplemented with fatty acid.. J Biochem.

[34] Fernandez-Valverde M, Reglero A, Martinez-Blanco H, Luengo JM (1993). Purification of *Pseudomonas putida* acyl coenzyme A ligase active with a range of aliphatic and aromatic substrates.. Appl Environ Microbiol.

[35] Garcia B, Olivera ER, Minambres B, Fernandez-Valverde M, Canedo LM (1999). Novel biodegradable aromatic plastics from a bacterial source.. J Biol Chem.

[36] Olivera ER, Carnicero D, Garcia B, Minambres B, Moreno MA (2001). Two different pathways are involved in the β-oxidation of *n*-alkanoic and *n*-phenylalkanoic acids in *Pseudomonas putida* U: genetic studies and biotechnological applications.. Mol Microbiol.

[37] Barber CE, Tang JL, Feng JX, Pan MQ, Wilson TJG (1997). A novel regulatory system required for pathogenicity of *Xanthomonas campestris* is mediated by a small diffusible signal molecule.. Mol Microbiol.

[38] Bajaj V, Lucas RL, Hwang C, Lee CA (1996). Co-ordinated regulation of *Salmonella typhimurium* invasion genes be environmental and regulatory factors is mediated by control of *hilA*.. Mol Microbiol.

[39] Cole ST, Brosch R, Parkhill J, Garnier T, Churcher C (1998). Deciphering the biology of *Mycobacterium tuberculosis* from the complete genome sequence.. Nature.

[40] Cox JS, Chen B, McNeil M, Jacob WR (1999). Complex lipid determines tissue-specific replication of *Mycobacterium tuberculosis* in mice.. Nature.

[41] Lucas RL, Lostroh CP, DiRusso CC, Spector MP, Wanner BL (2000). Multiple factors independently regulate *hilA* and invasion gene expression in *Salmonella enterica* serovar typhimurium.. J Bacteriol.

[42] Rindi L, Fattorini L, Bonanni D, Iona E, Freer G (2002). Involvement of the *fadD33* gene in the growth of *Mycobacterium tuberculosis* in the liver of BALB/c mice.. Microbiology.

[43] Soto MJ, Fernandez-Pascual M, Sanjuan J, Olivares J (2002). A *fadD* mutant to *Sinorhizobium meliloti* shows multicellular swarming migration and is impaired in nodulation efficiency on alfalfa roots.. Mol Microbiol.

[44] Campbell JW, Morgan-Kiss RM, Cronan JE (2003). A new *Escherichia coli* metabolic competency: growth on fatty acids by a novel anaerobic β-oxidation pathway.. Mol Microbiol.

[45] Iram SH, Cronan JE (2006). The β-oxidation systems of *Escherichia coli* and *Salmonella enterica* are not functionally equivalent.. J Bacteriol.

[46] Postle AD, Mander A, Reid KBM, Wang J-Y, Wright SM (1999). Deficient hydrophilic lung surfactant protein A and D with normal surfactant phospholipid molecular species in cystic fibrosis.. Am J Respir Cell Mol Biol.

[47] Brickman TJ, Vanderpool CK, Armstrong SK (2006). Heme transport contributes to *in vivo* fitness of *Bordetella pertussis* during primary infection in mice.. Infect Immun.

[48] Caiazza NC, Shanks RMQ, O'Toole GA (2005). Rhamnolipids modulate swarming motility patterns of *Pseudomonas aeruginosa.*. J Bacteriol.

[49] Krieg DP, Bass JA, Mattingly SJ (1988). Phosphorylcholine stimulates capsule formation of phosphate-limited mucoid *Pseudomoas aeruginosa*.. Infect Immun.

[50] Wargo MJ, Szwergold BS, Hogan DA (2008). Identification of two gene clusters and a transcriptional regulator required for *Pseudomonas aeruginosa* glycine betaine catabolism.. J Bacteriol.

[51] Bernhard W, Hoffmann S, Dombrowsky H, Rau GA, Kamlage A (2001). Phosphatidylcholine molecular species in lung surfactant: composition in relation to respiratory rate and lung development.. Am J Respir Cell Mol Biol.

[52] Hoffmann N, Rasmussen TB, Jensen PO, Stub C, Hentzer M (2005). Novel mouse model of chronic *Pseudomonas aeruginosa* lung infection mimicking cystic fibrosis.. Infect Immun.

[53] Mulcahy H, Charron-Mazenod L, Lewenza S (2010). *Pseudomonas aeruginosa* produces an extracellular deoxyribonuclease that is required for utilization of DNA as a nutrient source.. Environ Microbiol.

[54] Kang Y, Lunin VV, Skarina T, Savchenko A, Schurr MJ (2009). The long-chain fatty acid sensor, PsrA, modulates the expression of *rpoS* and the type III secretion *exsCEBA* operon in *Pseudomonas aeruginosa*.. Mol Microbiol.

[55] Hoang TT, Karkhoff-Schweizer RR, Kutchma AJ, Schweizer HP (1998). A broad-host-range Flp-*FRT* recombination system for site-specific excision of chromosomally-located DNA sequences: application for isolation of unmarked *Pseudomonas aeruginosa* mutants.. Gene.

[56] Hoang TT, Kutchma AJ, Becher A, Schweizer HP (2000). Integration-proficient plasmids for *Pseudomonas aeruginosa*: site-specific integration and use for engineering of reporter and expression strains.. Plasmid.

[57] Miller JH (1992). A short course in bacterial genetics..

[58] Hoang TT, Sullivan SA, Cusick KC, Schweizer PH (2002). *β*-Ketoacyl carrier protein reductase (FabG) activity of the fatty acid biosynthetic pathway is a determining factor of 3-oxo-homoserine lactone acyl chain lengths.. Microbiology.

[59] Bar-Tana J, Rose G, Shapiro B (1971). The purification and properties of microsomal palmitoyl-coenzyme A synthase.. Biochem J.

[60] Ichihara K, Shibasaki Y (1991). An enzyme-coupled assay for acyl-CoA synthase.. J Lipid Res.

[61] Wehrmann A, Vliet AV, Opsomer C, Botterman J, Schulz A (1996). The similarities of *bar* and *pat* gene products make them equally applicable for plant engineering.. Nat Biotechnol.

[62] Dowd JE, Riggs DS (1965). A comparison of estimates of Michaelis-Menten kinetics constant from various linear transformations.. J Biol Chem.

[63] Overhage J, Lewenza S, Marr AK, Hancock RE (2007). Identification of genes involved in swarming motility using a *Pseudomonas aeruginosa* PAO1 mini-Tn5-l*ux* mutant library.. J Bacteriol.

[64] Kouker G, Jaeger K-E (1987). Specific and sensitive plate assay for bacterial lipases.. Appl Environm Microbiol.

[65] Pinzon NM, Ju L-K (2009). Analysis of rhamnolipid biosurfactants by methylene blue complexation.. Appl Microbiol Biotechnol.

[66] Hoang TT, Schweizer HP (1997). Fatty acid biosynthesis in *Pseudomonas aeruginosa*: Cloning and characterization of the *fabAB* operon encoding *β*-hydroxyacyl-acyl carrier protein dehydratase (*fabA*) and *β*-ketoacyl-acyl carrier protein synthase I (*fabB*).. J Bacteriol.

[67] van den Berg B, Black PN, Clemons WM, Rapoport TA (2004). Crystal structure of the long-chain fatty acid transporter FadL.. Science.

[68] Holloway BW, Römling U, Tümmler B (1994). Genomic mapping of *Pseudomonas aeruginosa* PAO.. Microbiology.

[69] Yanisch-Perron C, Vieira J, Messing J (1985). Improved M13 cloning vectors and host strains: nucleotide sequences of the M13mp18 and pUC19 vectors.. Gene.

