# Correction: Multiple FadD Acyl-CoA Synthetases Contribute to Differential Fatty Acid Degradation and Virulence in *Pseudomonas aeruginosa*

**DOI:** 10.1371/annotation/a55051c6-25dc-4548-912e-bf339830668e

**Published:** 2010-11-17

**Authors:** Yun Kang, Jan Zarzycki-Siek, Chad B. Walton, Michael H. Norris, Patrick Videau, Mike Son, Tung T. Hoang

The authors requested additional authors be added to the byline and Author Contributions sections of this manuscript. The correct author byline (with affiliation footnotes) is: Yun Kang(2), Jan Zarzycki-Siek(1), Chad B. Walton(3), Michael H. Norris(2), Patrick Videau(1), Mike Son(1), Tung T. Hoang(1,2*).

The corrected citation is: Kang Y, Zarzycki-Siek J, Walton CB, Norris MH, Videau P (2010) Multiple FadD Acyl-CoA Synthetases Contribute to Differential Fatty Acid Degradation and Virulence in Pseudomonas aeruginosa. PLoS ONE 5(10): e13557. doi:10.1371/journal.pone.0013557

The correct Author Contributions are: Conceived and designed the experiments: YK CBW TTH. Performed the experiments: YK JZS MHN. Data analysis: YK TTH. Contributed reagents/materials/analysis tools: JZS CBW MHN. Wrote the paper: YK TTH. PV assisted JZS with the virulence assays. MS assisted YK with the enzyme assays.

